# Ultrastructure of coarse granules in the perivitelline space and association with ovulation induction protocols

**DOI:** 10.5935/1518-0557.20230032

**Published:** 2023

**Authors:** Sibel Bulgurcuoglu-Kuran, Ayse Altun, Fatma Nur Karakus, Tugba Kotil, Bilge Ozsait-Selcuk

**Affiliations:** 1 IVF Unit, Department of Obstetrics and Gynecology, Istanbul Faculty of Medicine, Istanbul University, Istanbul, Turkiye; 2 Department of Histology and Embryology, Cengiz Gokcek Women’s Health and Children Hospital, Gaziantep, Turkiye; 3 Department of Histology and Embryology, Istanbul Faculty of Medicine, Istanbul University, Istanbul, Turkiye; 4 Department of Medical Genetics, Istanbul Faculty of Medicine, Istanbul University, Istanbul, Turkiye

**Keywords:** oocyte quality, coarse granules in the perivitelline space, antagonist protocol, embryo, assisted reproduction

## Abstract

**Objective:**

To evaluate the origin and ultrastructure of the coarse granules in the perivitelline space (PVS) of oocytes of a group of couples attending assisted reproduction treatment.

**Methods:**

The ultrastructure of five oocytes with coarse granulues in the PVS obtained from three patients were evaluated by transmission electron microscopy (TEM). The influence of the ovulation induction regimen on the formation of granules in the PVS of the oocytes of 214 couples and the developmental capacity of these oocytes presenting granules in the PVS was analyzed retrospectively.

**Results:**

In TEM analysis, the microvilli structure was irregular, short, and loosely scattered through the oolemma in the oocytes presenting coarse granules in the PVS. Furthermore, dense lipid droplets were identified within the PVS and the surrounding cumulus cells. In retrospective analysis, the number of oocytes with coarse granules in the PVS was positively correlated with the duration of antagonist administration (r=0.23, *p*=0.013). Regardless of the type of granule, the presence of coarse or moderately coarse granules in the PVS was positively correlated with low-quality embryos on D3 (r=0.29, *p*=0.005) and the total number of arrested embryos up to D3 (r=0.33, *p*<0.001). Furthermore, the presence of coarse granules in the PVS severely exacerbated miscarriage rates.

**Conclusions:**

Our findings suggest that the presence of especially coarse granules in the PVS is correlated with the reduction of further embryonic developmental capacity in post-implantation stages of embryonic development, indicating a negative impact from aggressive ovulation induction protocols on developing oocytes.

## INTRODUCTION

A competent oocyte, imbued with the ability to develop into a healthy embryo after fertilization, resides in a highly orchestrated microenvironment of the ovarian follicle (Eppig, 1996; Revelli *et al*., 2009; Da Broi *et al*., 2018). Various factors such as genetic mutations, advanced maternal age, inappropriate ovulation induction protocols, or suboptimal culture conditions have been associated with oocyte quality, morphology, and subsequent embryo development (Ebner *et al.,* 2005; Rienzi *et al*., 2008; Pantasri & Norman, 2014; Ashrafi *et al.,* 2015). Although oocyte morphology is a predictor of embryo development and assisted reproduction treatment (ART) outcomes (Rienzi *et al*., 2008), these associations could not be verified in other studies (De Sutter *et al.,* 1996; Meriano *et al.,* 2001; Balaban & Urman, 2006; Chamayou *et al.,* 2006).

Besides the cytoplasmic morphologic features, an oocyte is expected to have a small perivitelline space (PVS) and no granules in the PVS (Veeck, 1988). Granules are among the extracytoplasmic morphologic abnormalities observed in 10.6% of human mature oocytes. They are categorized into two groups, coarse and moderately coarse granules (Farhi *et al.,* 2002; Balaban & Urman, 2006; Halvaei *et al.,* 2012). Controversial results have been reported on the influence of PVS size on embryo development and ART outcomes, and the effect of granules in the PVS was evaluated in a limited number of studies (Hassan-Ali *et al.,* 1998; Farhi *et al.,* 2002; Balaban & Urman, 2006; Chamayou *et al.,* 2006; Ten *et al.,* 2007; Coticchio *et al.,* 2016). Although there are studies reporting decreased fertilization rate, embryo quality, or implantation rates in the presence of granules in the PVS (Farhi *et al.,* 2002), other studies demonstrated no such outcomes (Xia, 1997; Hassan-Ali *et al.,* 1998; Ten *et al.,* 2007). It was previously suggested that coarse granules in the PVS might be associated with oocyte maturation defects and could be induced by elevated doses of human menopausal gonadotropins (hMG) as well as advanced maternal age and abnormal formation of the zona pellucida (Van Blerkom, 1990; Hassan-Ali *et al.,* 1998; Halvaei *et al.,* 2012). Postmaturation and in vitro aging of oocytes were also associated with granules in the PVS (Miao *et al.,* 2009). On the other hand, the presence of coarse granules in the PVS of the majority of the oocytes from different cycles of some patients (Farhi *et al.,* 2002) suggests a specific underlying mechanism.

The oocyte dysmorphologies evaluated in ART success predominantly involve cytoplasmic and perivitelline space size abnormalities (Alikani *et al.,* 1995; De Sutter *et al.*, 1996; Chamayou *et al.,* 2006). Few studies have investigated the effect of granules in the PVS alone (Hassan-Ali *et al.,* 1998; Farhi *et al.,* 2002; Hassa *et al.,* 2014). This study aimed to evaluate the origin and ultrastructure of coarse granules in the PVS in the oocytes of a group of couples undergoing ART treatment. Furthermore, the influence of the ovulation induction regimen was analyzed retrospectively.

## MATERIALS AND METHODS

### Patient selection

The ultrastructure of the five oocytes presenting coarse granules in the PVS obtained from three females undergoing ART at a university-based infertility clinic was evaluated by transmission electron microscopy (TEM). Only the oocytes that failed to fertilize after ICSI were included in the study.

The retrospective data of 121 couples undergoing ART between April 2008 and October 2018 at a university-based infertility clinic were analyzed to evaluate the association between granules in the PVS and ovulation induction protocol regimen and the influence of granulation on subsequent embryo development and ART outcomes. The inclusion criteria were presence of more than 60% of coarse or moderately coarse granules in the PVS of the oocytes of the patients. In order not to mask the results of the study, a control group was selected from ART patients whose oocytes presented no granules in the PVS or other morphologic abnormalities. The use of ART techniques other than ICSI (i), such as testicular sperm extraction (TESE) and frozen-TESE cycles, conventional in vitro fertilization cycles and frozen embryo transfer cycles, and the presence of any accompanying diseases other than infertility (ii) were the exclusion criteria of the study. Diagnoses of infertility of the study population were ovulatory factor (n=30), unexplained infertility (n=37), tubal factor (n=12) and male factor (n=42). The retrospective study population was analyzed in three groups depending on oocyte PVS morphology: presence of coarse granules in the PVS, (n=20, Group I), moderately coarse granules in the PVS (n=41, Group II), and no granules in the PVS (n=60, Group III, control group). This study was approved by the Ethics Committee of the institution (Date: 29.06.2018, No: 976).

### Controlled ovarian hyperstimulation and assisted reproduction techniques

The patients in the TEM group and the patients in the retrospective analysis group were treated using standard protocols. Controlled ovarian hyperstimulation (COH) was performed with standard gonadotropin hormone in an antagonist protocol. Recombinant FSH (Gonal F, Serono) was administrated daily starting from day three of the menstrual cycle. A fixed dose of GnRH antagonist (Cetrotide 0.25 mg/day; Serono, Geneva, Switzerland) was used from the sixth day of stimulation until the administration of human chorionic gonadotropin (hCG). Serum estradiol levels and ultrasound monitoring were used in the evaluation of follicular development. Transvaginal follicular aspiration was performed 36 hours after the administration of human chorionic gonadotropin (Ovitrelle, Merck KGaA, Darmstadt, Germany).

Three hours after oocyte pick-up, the oocytes were treated with hyaluronidase (Hyase 10×Vitrolife, Sweden), and their morphologies were evaluated under an inverted microscope (SZX16/SZX10, Olympus) equipped with Hoffmann modulation optics (magnification at X400). A binary scoring methodology (0 or I) was used for COC scoring, where a score of I is defined as having an expanded cumulus and a radiating corona (good quality) (Alpha Scientists in Reproductive Medicine and ESHRE Special Interest Group of Embryology, 2011). The PVS and the cytoplasmic and polar body morphologic abnormalities of the oocytes were recorded. All oocytes were fertilized using intracytoplasmic sperm injection (ICSI) and cultured individually in a sequential medium system (Vitrolife, Sweden) until day three of development (D3). Coarse granules in the PVS were defined as the presence of dark and dense granules in the PVS; moderately coarse granules in the PVS were defined as the presence of small inclusion-like debris in the PVS of the oocytes (Farhi *et al.*, 2002). The quality of the resulting embryos was graded daily according to the modified laboratory standards, in which Grade I and II (GI-II) represented good-quality embryos, and Grade III (GIII) featured low-quality embryos (Alpha Scientists in Reproductive Medicine and ESHRE Special Interest Group of Embryology, 2011). Depending on the laboratory protocols, embryo transfer (ET) was performed on D3. According to the current Turkish ART legislation, elective single or double ET was performed for females under or over 35 years, respectively.

In retrospective data analysis, we evaluated the association between the presence of coarse or moderately coarse granules in the PVS with 1) female age; 2) female BMI; 3) total dose of gonadotropins administered for ovarian hyperstimulation; 4) total dose of antagonists; 5) duration of antagonist administration (days); 6) fertilization rate; 7) embryo quality 68-72 hours after ICSI; and 8) ART outcomes.

### Transmission electron microscopy

The five oocytes obtained from failed fertilization cycles were fixed and processed for TEM analysis as described previously (Kotil *et al.,* 2018). Ultra-thin sections (60-80 nm) were examined on a Jeol JEM 1011 transmission electron microscope and photographed using the Soft Imaging System Analysis program with a Megaview III digital camera.

### Statistical analysis

The power analysis of the study was performed with G*Power version 3.1.9.7 (Faul *et al.,* 2007). Analysis of variance (ANOVA) was used to compare between the continuous variables of the three study groups. Tukey’s post-hoc test was used for multiple comparisons. Categorical variables such as implantation and pregnancy rates were compared using the chi-squared test. Correlation analysis was performed using the Spearman Rank Correlation Coefficient. A *p*-value <0.05 was considered statistically significant. Values were given as means and standard deviations (SD). All statistical analyses were performed on SPSS version 21.0 software (IBM Corp., Armonk, NY, USA).

## RESULTS

### Ultrastructure analysis of oocytes with granules in the PVS

**Figure 1:**
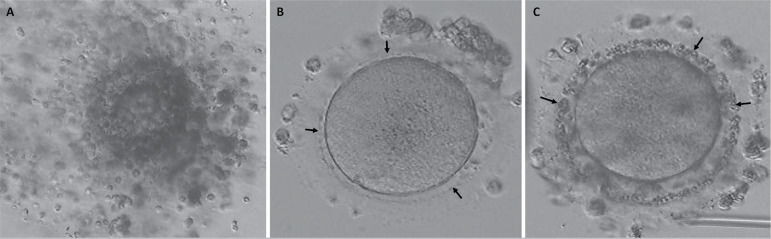
Oocytes with coarse and moderately coarse granules in the PVS. The oocytes with coarse granules in the PVS had significantly darker and dense cumulus cells (A) (40X magnification). Moderately coarse (B) and coarse (C) granules in the PVS (arrows) are observed in inverted microscopic evaluation of the oocytes after denudation at 100X magnification.

**Figure 2:**
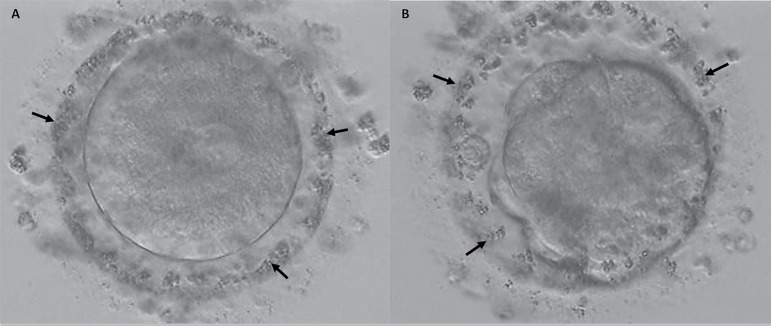
Embryonic development of an oocyte with coarse granules in the PVS. Coarse granules in the PVS (arrows) were still present at the pronuclear stage (A) and during the compaction of the oocytes (200X magnification).

The ultrastructure of five oocytes obtained from three failed fertilization cycles was evaluated by TEM. Patient characteristics are given in Supplementary Table 1. The oocytes with coarse granules in the PVS had significantly darker and dense cumulus cells than the oocytes with moderately coarse granules in the PVS (Figure 1), and had a low COC score (COC=0). Furthermore, the fertilized sibling oocytes obtained from these patients were monitored daily and their morphological characteristics were recorded. We observed that the coarse granules in the PVS were still present after fertilization and even at the cleavage stage embryos (Figure 2).

**Figure 3:**
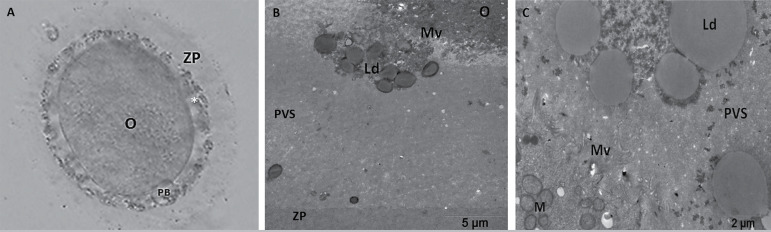
Microscopic and ultrastructural evaluation of an oocyte from patient 1. Coarse granules in the PVS can be clearly observed at 100X magnification with an inverted microscope (A). Lipid structures are observed either in association with microvilli (B) or dispersed within the PVS (C). O, oocyte; ZP, zona pellucida; PB, polar body; Mv, microvilli; PVS, perivitelline space; M, mitochondria; Ld, lipid droplet.

**Figure 4:**
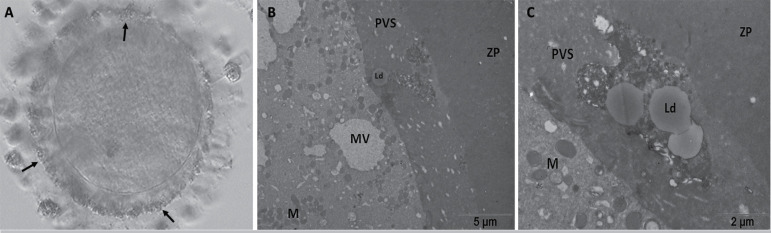
Microscopic and ultrastructural evaluation of an oocyte from patient 2. Coarse granules in the PVS can be clearly observed at 200X (A, arrow) magnification with an inverted microscope. ZP, zona pellucida; PVS, perivitelline space; M, mitochondria; MV, mitochondria-vesicle complex; Ld, lipid droplet.

After TEM evaluation, we observed that oocytes with coarse granules in the PVS and microvilli formation were irregular, short, and loosely scattered through the oolemma. The cortical granules were located close to the oolemma. Large smooth endoplasmic reticulum (ER) vesicles that were associated with mitochondria (mitochondria-vesicle complex, MV), small ER vesicles, and tubular aggregates were observed in the cytoplasm. Mitochondria were randomly distributed in the ooplasm, and their cristae formation could be detected. Dense lipid droplets were identified within the PVS and the surrounding cumulus cells (Figures 3 and 4).

### Results of retrospective data analysis

Data comprising 736 metaphase II (MII) and 88 immature (metaphase I (MI) or germinal vesicle (GV)) oocytes retrieved from 121 couples attending ART were evaluated retrospectively. The power of the study was calculated as 86%. In the overall group, the mean female age was 32.33 (±4.66); BMI 26.11 (±5.03); baseline FSH 7.44 (±3.29); baseline LH 6.86 (±5.82); and baseline estradiol 48.90 (±30.11). The characteristics of the three study groups are given in Table 1.

**Table 1:** Characteristics of the study groups.

Characteristics	Group I (coarse granules in the PVS) n=41	Group II (moderately coarse granules in the PVS) n=20	Group III (control group) n=60	*p*-value
**Age, years**	32.90 (±5.08)	31.83 (±4.21)	32.48 (±4.85)	0.661
**BMI, kg/m^2^**	27.53 (±5.79)	25.98 (±4.26)	25.72 (±5.24)	0.375
**FSH, mIU/mL**	8.10 (±5.56)	7.02 (±2.35)	7.51 (±2.84)	0.477
**LH, mIU/mL**	6.71 (±2.90)	6.96 (±3.71)	6.85 (±7.53)	0.987
**E2, pg/mL**	51.72 (±23.57)	54.47 (±36.81)	53.16 (±27.43)	0.944
**Total gonadotropin dose, IU/ml**	2530.58 (±1204.63)	2303.54 (±658.93)	2359.02 (±707.53)	0.574
**Total antagonist dose, mg**	1.21 (±0.35)	1.06 (±0.28)	1.03 (±0.35)	0.085
**Duration of antagonist administration, days), n**	4.85 (±1.39)	4.21 (±1.13)	4.10 (±1.39)	0.085
**MII, n**	5.90 (±5.14)	6.76 (±4.23)	5.65 (±3.39)	0.397
Immature oocytes^*^, n	0.60 (±0.99)	0.90 (±1.32)	0.65 (±1.00)	0.464
**Fertilization, %**	62.98 (±33.92)	66.63 (±29.79)	72.67 (±23.36)	0.317
**D3 good-quality embryos, n**	2.41 (±3.18)	3.64 (±3.03)	3.84 (±2.45)	0.193
**D3 low-quality embryos, n**	0.24 (±0.56)	0.54 (±0.99)	0.04 (±0.21)	0.006
**Arrested Embryos, n**	0.59 (±1.23)	0.55 (±0.81)	0.00	< 0.001
**Implantation (yes), %**	16.7	22.9	23.3	0.830
**Clinical pregnancy, (yes) %**	16.7	22.9	23.3	0.830
**Abortus, (yes), %**	16.7	5.7	1.7	0.045
**Live birth, (yes), %**	0	75.0	85.7	0.011

Values are expressed as mean ± standard deviation (SD). P values are obtained by ANOVA or chi-squared tests. A *p*-value < 0.05 is considered statistically significant. Hormone levels represent the baseline level measured on day three of menstruation. BMI, body mass index; FSH, follicle stimulating hormone; LH, luteinizing hormone; E2, estradiol; MII, metaphase II; D3, developmental day three.

* Immature oocytes include both germinal vesicle and metaphase I oocytes. #Arrested embryos represent the total number of arrested embryos up to day three of development.

The mean BMI in the group with coarse granules in the PVS was higher than that of the other groups, though not statistically significant (*p*=0.375). To investigate whether the presence of coarse or moderately coarse granules in the PVS was influenced by female BMI, the overall study group was stratified according to BMI level (cut-off level=25 kg/m^2^). We observed that the likelihood of the presence of coarse or moderately coarse granules in the PVS was higher when female BMI >25 kg/m^2^ (*p*=0.025). On the other hand, in women with lower a BMI, total antagonist dose and days of antagonist administration were significantly higher in the group with coarse granules in the PVS than in the group with moderately coarse granules in the PVS and in the control group (*p*=0.004 and *p*=0.004, respectively; Table 2).

**Table 2:** Characteristics of the study group when stratified according to BMI (cut-off level=25 kg/m^2^).

	BMI<25 m^2^/kg				BMI>25 m^2^/kg			
	**Coarse PVSi**	**Moderate PVSi**	**Control group**	***p*-value**	**Coarse PVSi**	**Moderate PVSi**	**Control group**	***P*-value**
**Age**	33.00 (±5.59)	31.77 (±5.12)	31.40 (±4.23)	0.742	32.86 (±5.07)	31.77 (±3.94)	33.37(±5.14)	0.458
**FSH, mIU/mL**	10.80 (±9.19)	7.76 (±2.29)	7.99 (±3.31)	0.293	6.95 (±2.81)	6.79 (±2.39)	7.10 (±2.33)	0.902
**LH, IU/mL**	7.13 (±2.77)	6.17 (±2.36)	8.11 (±10.30)	0.778	6.52 (±3.04)	7.18 (±4.19)	5.59 (±2.58)	0.236
**E2, pg/mL**	47.15 (±25.20)	54.25 (±25.67)	56.88 (±24.01)	0.673	53.68 (±23.53)	55.22 (±42.22)	49.50(±31.65)	0.831
**Total gonadotropin dose, IU/ml**	2968.66 (±1206.28)	2309.23 (±552.77)	2351.77 (±567.98)	0.103	2342.82 (±1197.78)	2278.85 (±734.79)	2331.96 (±864.92)	0.968
**Total antagonist dose, mg**	1.38 (±0.21)	1.04 (±0.23)	0.98 (±0.27)	0.004	1.14 (±0.38)	1.05 (±0.31)	1.07 (±0.42)	0.732
**Duration of antagonist administration (days), n**	5.50 (±0.84)	4.15 (±0.90)	3.90 (±1.09)	0.004	4.57 (±1.51)	4.19 (±1.23)	4.26 (±1.68)	0.732
**MII, n**	5.17 (±2.48)	5.77 (±3.79)	5.13 (±2.26)	0.780	6.21 (±5.99)	7.42 (±4.65)	6.07 (±4.13)	0.552
**Immature oocytes, n**	1.17 (±1.60)	0.85 (±1.35)	0.60 (±0.97)	0.512	0.72 (±1.12)	0.96 (±1.37)	0.67 (±1.07)	0.261
**Fertilization, %**	62.83 (±30.11)	67.62 (±35.02)	78.07 (±22.34)	0.311	63.04 (±36.51)	65.50 (±27.81)	66.62 (±24.64)	0.930
**D3 good-quality embryos, n**	2.33 (±1.97)	3.25 (±3.11)	3.96 (±1.83)	0.248	2.45 (±3.78)	3.89 (±3.22)	3.37 (±2.69)	0.347
**D3 low-quality embryos, n**	0.17 (±0.41)	0.75 (±1.49)	0.08 (±0.28)	0.088	0.27 (±0.65)	0.50 (±0.79)	0.00	0.500
**Arrested Embryos#, n**	1.00 (±2.00)	0.45 (±0.82)	0.00	0.014	0.31 (±0.48)	0.50 (±0.80)	0.00	0.037

Values are expressed as mean ± standard deviation (SD). *P* values are obtained by the ANOVA test. A *p*-value <0.05 is considered statistically significant. Hormone levels represent the basal level measured on day three of menstruation. BMI, body mass index; FSH, follicle stimulating hormone; LH, luteinizing hormone; E2, estradiol; MII, metaphase II; D3, developmental day three.

* Immature oocytes include both germinal vesicle and metaphase I oocytes. #Arrested embryos represent the total number of arrested embryos up to day three of development.

### Coarse granules in the PVS were induced by the ovulation induction regimen

The total gonadotropin dose used in the ovulation induction protocol was not statistically different among the study groups (*p*=0.574) (Table 1). Though not statistically significant, the mean antagonist dose was higher in the group with coarse granules in the PVS than in the group with moderately coarse granules in the PVS and in the control group. However, in the non-parametric correlation test, the number of oocytes with coarse granules in the PVS was positively correlated with the total antagonist dose (r=0.23, *p*=0.013) and the duration of antagonist administration (r=0.23, *p*=0.013).

### Coarse granules in the PVS affect embryo development and ART outcomes

The number of oocytes presenting coarse granules in the PVS (*p*=0.585) or moderately coarse granules in the PVS (*p*=0.499) was not comparable among patients with different infertility diagnoses. Although the mean fertilization rate was lower in the group with coarse granules in the PVS than in the other two groups, the difference was not statistically significant (*p*=0.317). Presence of coarse granules in the PVS was negatively correlated with the number of good-quality embryos on D3 (r=-0.271, *p*=0.010). Regardless of type, the presence of coarse or moderately coarse granules in the PVS was positively correlated with low-quality embryos on D3 (r=0.29, *p*=0.005) and the total number of arrested embryos up to D3 (r=0.33, *p*<0.001).

To examine the impact of granules in the PVS on the quality and competency of the developed embryos, we compared the ART outcomes among the study groups. Interestingly, we found that the presence of granules in the PVS had no impact on implantation (*p*=0.830) or clinical pregnancy rates (*p*=0.830). However, presence of coarse granules in the PVS was associated with a high rate of miscarriages (*p*=0.045) (Table 1). The live birth rate of the group with moderately coarse granules in the PVS was 75.0%, while in controls the rate was 87.5%; None of the clinical pregnancies from oocytes with coarse granules in the PVS reached term, with miscarriages occurring early in the first trimester (*p*=0.011).

## DISCUSSION

Although controversial, excessive ovarian response and gonadotropin overdose were associated with lower quality oocytes with a higher incidence of intracytoplasmic defects and presence of granules in the PVS (Hassan-Ali *et al.,* 1998; de Cássia S Figueira *et al.,* 2010). Our study showed that coarse granules in the PVS have a distinct ultrastructure associated with lipid structures. We found that not gonadotropin overdose, but the antagonist protocol regimen, might influence oocyte quality and further embryo development. Furthermore, the presence of coarse granules in the PVS severely exacerbated miscarriage rates.

It has been suggested that coarse granules in the PVS originate from the ooplasm, prolongations of corona radiate cells, or protrusions of cumulus cells that entered the PVS due to zona pellucida abnormalities (Dandekar *et al.,* 1992; Sathananthan, 1997; Rankin *et al.,* 1999). A mature oocyte with normal morphology has numerous long and thin microvilli extending into the PVS (Palmerini *et al.,* 2014). However, in aged oocytes, microvillar protrusions show structural alterations and bud-off into the PVS (Miao *et al.,* 2009; Bianchi *et al.,* 2015). In the ultrastructure analysis of the oocytes with coarse granules in the PVS, we observed that the distribution and morphology of the microvilli were altered when compared to previously reported data (Dandekar *et al.,* 1992; Sathananthan, 1997; Rankin *et al.,* 1999; Palmerini *et al.,* 2014). The cortical granules were located near the oolemma, and several MVs were apparent in the ooplasm. Normally, cortical granules and MVs are not expected to be present after oocyte activation and fertilization (El Shafie *et al.*, 2000). The presence of these structures after ICSI suggests that fertilization failed possibly due to a lack of oocyte activation.

Interestingly, we observed that lipids contribute to the formation of coarse granules in the PVS also located in the cumulus cell compartment. To investigate the possible effect of BMI on the formation of coarse granules in the PVS, we stratified the retrospective data according to BMI (cut-off level=25 kg/m^2^) and found that likelihood of having coarse or moderately coase granules in the PVS was higher when female BMI was greater than 25 kg/m^2^. However, the BMI alone did not explain the high proportion of embryonic arrest in the group with coarse granules in the PVS. Interestingly, we observed that the total antagonist dose and the duration of the antagonist protocol were significantly associated with the presence of coarse granules in the PVS, not in the higher but in the lower BMI group. This finding highlights the significant impact of aggressive ovulation induction on oocyte development and oocyte dysmorphisms in relation to BMI.

In previous studies, Hassan-Ali *et al*. (1998) showed a significant increase in PVS granulation in cases of excessive HMG administration. Hassan-Ali *et al*. (1998) and de Cássia S Figueira *et al*. (2010) suggested a negative influence on oocyte quality with excessive ovulation induction protocols (Farhi *et al.,* 2002; de Cássia S Figueira *et al.,* 2010). Similarly, we observed that especially prolonged antagonist induction was associated with the presence of coarse granules in the PVS in our study population. These results suggest that an aggressive ovulation induction regimen might induce the formation of coarse granules in the PVS. This dysmorphism might arise by the disruption of the prolongations of the corona radiate or cumulus cells in the PVS of the developing oocyte affected by an unfavorable follicular environment. As a result, the competency of the oocyte to further develop into a higher-quality embryo might be hampered.

In the evaluation of the morphology of the developing embryos among study groups, we observed that coarse granules in the PVS were persistent in the cleavage stage embryos, as also reported in previous studies (Farhi *et al.,* 2002; Halvaei *et al.,* 2012). In their study, Hassan-Ali *et al*. (1998) reported that coarse granules in the PVS were not correlated with fertilization rates or embryo development (Farhi *et al.,* 2002; Ten *et al.,* 2007). However, the authors demonstrated that PVS abnormalities such as a large perivitelline space with or without granules might influence embryo development (Hassa *et al.,* 2014). In our study population, although fertilization rates were not comparable between the selected controls and groups with granules in the PVS, coarse granules in the PVS negatively affected the quality of cleavage stage embryos and induced early embryonic arrest. When the ART outcomes were analyzed, one study demonstrated that the presence of coarse granules in the PVS was correlated with lower implantation and ongoing pregnancy rates in ART cycles (Farhi *et al.,* 2002). Conversely, other studies found no correlation between ART outcomes and coarse granules in the PVS accompanied by either PVS size abnormality or coarse granules in the PVS alone (Hassan-Ali *et al.,* 1998; Chamayou *et al.*, 2006). In our study, although implantation and clinical pregnancy rates were not different between the groups with granules in the PVS and controls, the miscarriage rate was significantly higher in the group with coarse granules in the PVS.

The size of the population is a limitation of our study. However, in order not to mask the results of this specific dysmorphism, we only included patients whose oocytes had only granules in the PVS as a dysmorphism and presented no other PVS or cytoplasmic abnormality. In a similar approach, only the oocytes with normal morphology were included in the control group. The control group included top quality oocytes and, probably due to the strict criteria of the study, no cases of embryonic arrest were observed in the control group and none of the embryos were miscarried after implantation. And this is another limitation that comes from the design of the study itself. Similar studies with larger populations might overcome such limitations.

In conclusion, there are contradicting results on the influence of coarse granules in the PVS on embryo quality and ART outcomes. However, taking the data together with our findings, we suggest that the presence of granules in the PVS and especially coarse ones is an oocyte dysmorphism that indicates an oocyte maturation defect and is correlated with decreased embryonic developmental capacity in post-implantation stages of embryonic development.

### Ethics statements

This study was approved by the Ethics Committee of Istanbul University Faculty of Medicine (Date: 29.06.2018, No: 976). The patients/couples provided their written informed consent to participate in this study.

## Appendix

**Supplementary Table 1:** The characteristics of the patients whose oocytes were histologically evaluated.

Characteristics	Patient 1	Patient 2	Patient 3
**Age, years**	37	38	28
**BMI, kg/m^2^**	27	25	29
**FSH, mIU/mL**	7.87	33	6.2
**LH, mIU/mL**	4.22	13.3	14
**PRL, ng/L**	7.27	7	49
**E2, pg/mL**	63.81	5	24

BMI, body mass index; FSH, follicle stimulating hormone; LH, luteinizing hormone; PRL, prolactin hormone; E2, estradiol; Hormone levels were measured on day three of menstruation.
